# Bromodomain AAA+ ATPases get into shape

**DOI:** 10.1080/19491034.2020.1741304

**Published:** 2020-03-19

**Authors:** Magdalena Murawska, Andreas G. Ladurner

**Affiliations:** Biomedical Center, Physiological Chemistry, Ludwig-Maximilians-University of Munich, Planegg-Martinsried, Germany

**Keywords:** Abo1, bromodomain AAA+ ATPase, histone chaperone

## Abstract

Bromodomain AAA+ ATPases (ATPases associated with diverse cellular activities) are emerging as oncogenic proteins and compelling targets for anticancer therapies. However, structural and biochemical insight into these machines is missing. A recent study by Cho *et al*. reports the first cryo-EM structure of a bromodomain AAA+ ATPase and provides first insights into the functions of this putative histone chaperone.

Bromodomain AAA+ ATPases are poorly characterized members of a large family of molecular machines, which utilize the energy of ATP to remodel a wide range of client proteins and nucleic acids [1]. *S. pombe* Abo1 is a highly conserved homologue of human ATAD2, which is an oncogene overexpressed in many cancers with poor prognosis [2]. ATAD2 is proposed to function as transcriptional co-factor of several oncogenic transcription factors, including Myc, estrogen and androgen receptors [2]. Abo1/ATAD2 is also a putative histone chaperone [3,4], it is linked to DNA replication [5], heterochromatin boundary maintenance [6,7] and transcription programs in ES cells [8]. Abo1 and ATAD2 can thus be added to a range of chromatin-interacting proteins of relevance in cancer biology, yet little is known about their function in mechanistic terms.

Despite these biological links, the regulation of these bromodomain AAA+ ATPases, molecular mechanism and substrate specificity are, in fact, largely unknown. A recent study from Song’a lab [9] now reports the first cryo-EM structure of Abo1, providing first biochemical glimpses on the potential role of Abo1 in mediating histone-DNA interactions.

The authors purified recombinant Abo1 from insect cells and characterized the enzyme biochemically. They showed that Abo1 is a hexamer regardless of the presence or the absence of ATP cofactor. Fluorescence anisotropy assays demonstrated that Abo1 binds recombinant histones H3-H4 with nanomolar affinity, in an interaction that also appears to be independent of nucleotides. Thus, in the basic (non-nucleotide bound state) Abo1 acquires an oligomeric state and binds to histone substrates *in vitro*.

The team then resolved several cryo-EM structures of Abo1 in the presence of ATP, ADP or in an apo state. For this, they used an ATP-hydrolysis-deficient mutant in the Walker B motif of the AAA1 domain. The conserved AAA domain of Abo1 contains two subdomains: AAA1 with two major nucleotide-binding (Walker A) and hydrolysis motifs (Walker B), and the AAA2 domain, which is presumed to be catalytically inactive. The structure confirmed the presence of a hexameric ring, where AAA1 domains form the top layer and AAA2 domains the bottom layer of the ring. The overall core structure of the ATPase domains of Abo1 is thus similar to the classical clade of AAA+ ATPases [1], but also exhibits several unique features. The subunits of the ring are connected through a molecular clasp, where the helix-turn-helix insert within the AAA2 nucleotide binding domain (NBD) of one subunit locks into a ‘hole’ formed by the AAA1 and AAA2 helical bundle domains (HBD), as well as a linker that proceeds the AAA2 HBD of the other subunit. This unique locking system explains most probably why Abo1 exists as a stable hexamer in solution.

Moreover, the authors observed nucleotide-dependent structural changes of Abo1 in the presence of ATP. In the apo and ADP states, the subunits form a symmetric ring, whereas in the ATP state, the subunits are shifted, forming a more asymmetric, opened spiral with a smaller pore and two subunits that are separated by a distance of 40 Å (Figure 1). In order to further explore these conformational changes, high-speed atomic force microscopy (HS-AFM) was used to follow dynamics of Abo1 in real time. This analysis revealed that the opening of Abo1 ring occurs randomly, which indicates that Abo1 subunits could hydrolyze ATP stochastically.

**Figure 1.**
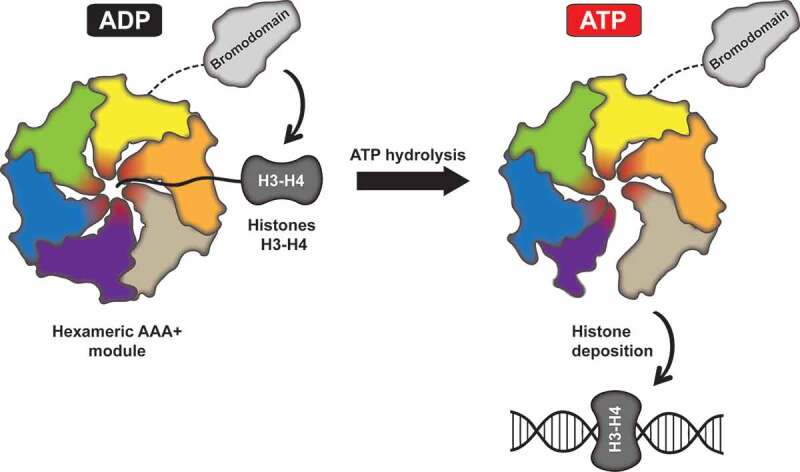
A putative model of histone deposition on DNA by Abo1/ATAD oncogenes. In the ADP state, Abo1 adopts a close hexameric ring shape. A negatively charged pore (marked by red color) can contact the N-terminal tails of histone H3. ATP binding and hydrolysis leads to conformational rearrangements of the subunits, causing Abo1 to assume a more open, spiral shape. ATP hydrolysis is essential for deposition of histones H3-H4 onto DNA. In addition, the protein’s bromodomains get in contact with histones and are important for the deposition reaction, which may occur through the tethering of histone substrates.

Finally, Cho et al. provide some biochemical evidence that Abo1 might be an unusual ATP-dependent histone chaperone. A single-molecule DNA curtain assay with labeled histones showed that Abo1 can deposit histones H3-H4 on DNA, but only in the presence of ATP (Figure 1). The electrostatic surface representation of Abo1 possesses a negatively charged pore. Alanine substitutions of two conserved pore residues, Trp 345 or Glu 385, abolished histone deposition in the DNA curtain assay without greatly affecting histone binding or ATP hydrolysis rates of Abo1. A crosslinking-mass spectrometry experiment suggested crosslinks between the H3 N-terminal tail and Abo1 pore residues. In addition, removal of histone tails by partial trypsinization abolished their deposition onto DNA. These assays thus suggest that histone N-terminal tails and the negatively charged pore of Abo1 might be important for the reported histone deposition activity of the Abo1 enzyme.

Despite good resolution of the AAA+ ATPase ring of Abo1, the cryo-EM structures published by Song’s lab lack the defining feature of these ATPases, namely the presence of the globular bromodomain module. In the stable apo state, a low-resolution structure suggested the possibility of the bromodomains forming a hexameric ring above the ATPase ring. In addition, two long linker regions joining the AAA+ and bromodomains were observed, which suggests potential flexibility of the bromodomains. Further research will be necessary to optimize sample preparation in order to obtain the full picture of how the bromodomains of Abo1/ATAD2 contribute to the overall structure and function of these enzymes. Overall, the study by Song and colleagues provides the first structural and biochemical insights into the poorly understood bromodomain AAA+ ATPase family. The work clearly shows that the *S. pombe* homologue, Abo1, is a stable hexamer and suggests a first model of how Abo1 might be involved in histone deposition on DNA (Figure 1).

The study raises the question about the biological function(-s) of bromodomain AAA+ ATPases. The authors utilized single-molecule DNA curtain assays to study histone deposition by Abo1. However, MNase protection assays with the Abo1 assembly products produced different DNA fragments than the tetrasomes generated by a *bona fide* histone chaperone such as CAF1. This suggests that Abo1 might generate some histone-DNA intermediates that require other factors to complete the assembly task. Interestingly, Abo1 was shown to be synthetic lethal with a mutant of the conserved and essential histone chaperone FACT [3]. This suggests potential cooperation between the two factors. It would be interesting to test whether nucleosome assembly by FACT is stimulated in the presence of Abo1.

In metazoans, the bromodomains of the related ATAD2 protein were shown to bind acetylated histones, although their substrate specificity is still not well established [5]. All the experiments performed by Cho et al. were done in the presence of unmodified histones. However, a predicted histone-binding mutant in the bromodomain of Abo1 inhibited histone deposition on DNA. This suggests that the ATAD2 bromodomains may play a role in histone substrate binding that is largely independent of their acetylation. It will be important to test more directly whether the acetylation of histones alters Abo1/ATAD2 binding to histones, ATPase activity and/or histone deposition.

Finally, the *bona fide* substrates of bromodomain AAA+ ATPases are still not known. In this regard, mass spectrometry of endogenous histones bound to Abo1/ATAD2 might reveal the specific histone modification(s) recognized by this protein. Moreover, since Abo1/ATAD2 co-purifies with several chromatin complexes [3,8], it is possible that similarly to Rvb AAA+ ATPases, Abo1 and its homologs participate in the assembly and/or remodeling activities of other chromatin bound complexes [10].

The research published by Song’s team provides the first step in understanding at the molecular and structural level this important group of oncogenic proteins, a first step in guiding the design and development of drugs targeting bromodomain AAA+ ATPases.
